# The Doctor’s Tax

**Published:** 1897-11

**Authors:** 


### The Doctor's Tax.

THE JOURNAL is in receipt of the following. See our editorial on this subject.

CORSICANA, Texas, Oct. 19, 1897.

Dear Doctor: At a meeting of the physicians of Navarro county, Texas, which was recently held in Corsicana, the object of which was to discuss the occupation tax recently imposed upon the medical profession, the following resolutions were read and after discussion unanimously adopted.

Resolved that we, the physicians of Navarro county, in convention assembled, not wishing lightly to question or antagonize the laws of our state, but deeply feeling that a wrong has been done our noble profession by the last legislature in the imposition of an occupation tax, will do all in our power to defeat the law or cause its repeal at the earliest hour.

For many years the policy of the State, guided by the wisdom of her great statesmen, has declared against such a tax.

The occupation tax is a levy upon such trades, occupations and callings as are supposed to be devoid of charitable or benevolent purpose for the benefit of the persons pursuing them.

The tax is supposed to be a return to the public for the privilege of engaging in the business and enjoying its remuneration.

Tested by this principle, the tax on physicians becomes unjust.

There is no profession upon whom more demands for charity are made, whose professional labor is so poorly paid, or more incessant and trying.

At all hours of the night and day, standing by the sick bed battling with disease and epidemics in every form, comforting the despairing and carrying light and hope to many a home and heart, it surely can challenge comparison with every other untaxed profession or institution benevolent or otherwise in this State, and justly claim that its public labor for public good, its vast and constant charities entitle it to exemption from this tax.

It is therefore resolved that we take steps to arouse our brethren in the State to an active, organized opposition in every county in the State. That we make test cases of the law, and in case of failure exert our influence before the next legislature to repeal it.

At the close of the meeting, a committee was appointed to confer with other members of the profession, requesting their co-operation in organizing to defeat or have repealed this tax, which is not such a great pecuniary imposition, but which involves principles that outweigh the tax which is imposed.

It is not the individual doctor who is insulted, but 'tis the medical profession at which the law strikes a blow, and thus seeks to turn one of the noblest of professions into a trade. The State of Texas thus emphatically declares that she is under no obligatiion to her medical profession.

Hoping to have your co-operation in this movement, which is of such vital importance to us all, we remain,

Fraternally yours,

A. C. Sloan,
W. J. W. Kerr,
A. O. Buck,
M. B. Pitts,
I. N. Suttle.
Committee.

